# Possible transmission of *Sarcoptes scabiei* between herbivorous Japanese serows and omnivorous Caniformia in Japan: a cryptic transmission and persistence?

**DOI:** 10.1186/s13071-019-3630-5

**Published:** 2019-08-05

**Authors:** Ryota Matsuyama, Toshihiro Yabusaki, Natsuko Senjyu, Tsukasa Okano, Minoru Baba, Tomoka Tsuji-Matsukane, Mayumi Yokoyama, Nobuhide Kido, Teruki Kadosaka, Takuya Kato, Masatsugu Suzuki, Makoto Asano

**Affiliations:** 1 Graduate School of Biomedical and Health Sciences, Hiroshima University, Hiroshima, Japan; 2 Faculty of Applied Biological Sciences, Gifu University, Gifu, Japan; 3 Gifu Prefectural Chuo Meat Inspection Office, Ogaki, Japan; 4 Kitakyushu Museum of Natural History and Human History, Kitakyushu, Japan; 5 Wildlife Research & Consulting Services Ltd., 94-2, Tanba, Japan; 6 Wildlife Management Research Center, Tanba, Hyogo Japan; 7 Zoorasia Yokohama Zoological Gardens, Yokohama, Japan; 8 Department of Infection and Immunity, Aichi Medical University, Nagakute, Japan; 9 Faculty of Veterinary Science, Nippon Veterinary and Life Science University, Musashino, Japan

**Keywords:** Host–parasite relationship, Host specificity, Sarcoptic mange, Scabies, Genetic structure

## Abstract

**Background:**

Two transmission patterns of *Sarcoptes scabiei* in host mammal communities have been reported based on microsatellite-level genetic studies in the last two decades. While one involves restrictions among different host taxa, the other is associated with predator–prey interactions between different host taxa. In contrast to these observations, the present study reports a possible irregular case of transmission of *S. scabiei* between herbivorous Japanese serow and omnivorous Caniformia mammals in Japan, though under very weak predator–prey relationships.

**Methods:**

DNA from 93 *Sarcoptes* mites isolated from omnivorous Caniformia (such as the domestic dog, raccoon dog, raccoon and Japanese marten), omnivorous Cetartiodactyla (wild boar) and herbivorous Cetartiodactyla (Japanese serow) in Japan were analyzed by amplifying nine microsatellite markers. Principal components analyses (PCA), Bayesian clustering analyses using STRUCTURE software, and phylogenetic analyses by constructing a NeighborNet network were applied to determine the genetic relationships among mites associated with host populations.

**Results:**

In all the analyses, the genetic differentiation of *Sarcoptes* mites from wild boars and Japanese serows was observed. Conversely, considerably close genetic relationships were detected between Caniformia-derived and Japanese serow-derived mites. Because the predator–prey interactions between the omnivorous Caniformia and herbivorous Japanese serow are quite limited and epidemiological history shows at least a 10-year lag between the emergence of sarcoptic mange in Japanese serow and that in Caniformia, the transmission of *S. scabiei* from Caniformia to Japanese serow is highly suspected.

**Conclusions:**

The close genetic relationships among mites beyond Host–taxon relationships and without obvious predator–prey interactions in Caniformia and Japanese serow deviate from previously reported *S. scabiei* transmission patterns. This type of cryptic relationship of *S. scabiei* populations may exist in local mammalian communities worldwide and become a risk factor for the conservation of the remnant and fragmented populations of wild mammals.

**Electronic supplementary material:**

The online version of this article (10.1186/s13071-019-3630-5) contains supplementary material, which is available to authorized users.

## Background

*Sarcoptes scabiei* is a notorious pathogenic mite species (Acari: Astigmata: Sarcoptidae) that causes a highly contagious dermatitis known as sarcoptic mange in mammalian hosts [1]. Among the mammalian species, and even within the species [2], the pathogenicity of the disease spans largely from devastating impacts on naive host individuals [1, 3] to relatively low pathogenic infections in less-vulnerable host individuals [4]. The impact of sarcoptic mange on mammalian host populations changes among populations and in the trade-offs among biological factors such as population density, host mortality rate and host reproductive rate [5–7]. However, this has often been viewed as a possible threat for the conservation of remnant and isolated mammal populations [1]. Although emerging (or re-emerging) outbreaks of sarcoptic mange in mammalian populations have been reported worldwide [1, 8], neither the origin of the epidemic lineages of *S. scabiei* nor the patterns of inter-species transmission of mites from the reservoir host species to the novel (or unobserved) host species have been sufficiently understood [9]. This is because the existence of a classical problem in the identification of mite strains, at least until the late 1990s, due to the nature of *S. scabiei* populations having different patterns of Host-specificities with indistinguishable morphology [8, 10]. To resolve this problem, a method for genetic analysis using microsatellite (simple sequence repeat; SSR) markers was developed by Walton et al. [11–13] and, since then, this technique has been utilized as an efficient way to elucidate the hidden transmission web of *Sarcoptes* mites in the multi-host system [9, 14].

Recent genetic studies using the SSR markers revealed two patterns of inter-species transmission of *Sarcoptes* mites: one is the transmission restricted by the “host taxon” [15–17] and the other is the transmission through “prey–predator” interaction [18–20]. For Host–taxon transmission restriction, Rasero et al. [15] found that *Sarcoptes* mite populations from ten European wild mammal species were genetically separated by three patterns of host taxon, named as carnivores, omnivores and herbivores (in the definition of Rasero et al. [15]). For predator–prey transmission, close genetic associations between mite populations derived from predators and their preferred prey were detected, e.g. between lion (*Panthera leo*) and wildebeest (*Connochaetes taurinus*), and between cheetah (*Asinonyx jubatus*) and Thomson’s gazelle (*Gazelle thompsonii*) [18]. The difference between these two patterns of transmission of *Sarcoptes* mites suggests the existence of diversity in their transmission web, associated with the interaction within the local mammalian communities co-existing in each region [9]. In Japan, the realization of the epidemic of sarcoptic mange in wild mammal hosts was started in early 1980s with the report of an outbreak in raccoon dogs (*Nyctereutes procyonoides*) [21, 22]. Since then, during the past 40 years, the disease has been drastically expanded in the omnivorous Carnivora species [e.g. red fox (*Vulpes vulpes*), masked palm civet (*Paguma larvata*)] and wild boars (*Sus scrofa leucomystax*) [21–23]. In addition, cases of infestation of *Sarcoptes* mites in Japanese serow (*Capricornis crispus*), an indigenous ruminant species designated as a Special National Monument in Japan, have been reported since the mid-1990s [21, 24, 25]. Because of the nearly simultaneous (within 10–20 years) and multiple beginnings of epidemics in wild mammals, it has been suspected that possible inter-species transmission occurred in these mammalian host communities, with the addition of transmission from domestic/companion animals (e.g. dog, swine and cattle) [21, 22]. However, the genetic relationships of *Sarcoptes* mites with mammalian hosts have not been elucidated at the level of SSR resolution, apart from limited reports on the relationship between raccoon dog populations and domestic/companion dogs (*Canis familiaris*) [26].

In the present study, we aim to illuminate the neglected relationships of *Sarcoptes* mite populations derived from five wild mammalian species and domestic/companion dogs in Japan by SSR analysis. This enables us to explicate whether the transmission within these multi-host species follows the host taxon and predator–prey patterns. The target hosts were medium-sized omnivorous Carnivora, more specifically Caniformia [domestic dog, raccoon dog, raccoon (*Procyon lotor*) and Japanese marten (*Martes melampus melampus*)], an omnivorous Cetartiodactyla (wild boar) and a herbivorous Cetartiodactyla (Japanese serow).

To our knowledge, the predator–prey relationships between these omnivorous Caniformia and Cetartiodactyla have not been previously reported, except for a few limited cases of predation of Japanese serow by feral dogs [27] and possible occasional scavenging on carcasses of omnivorous species [28–31]. Against these inadequate predator–prey relationships and the expected Host–taxon barriers for the inter-species transmission, we report here the observation of “cryptic,” close genetic relationships among the *Sarcoptes* mites derived from Japanese serows and those from omnivorous species in Caniformia.

## Methods

### Collection of *S. scabiei* from skin crusts of hosts

**Fig. 1:**
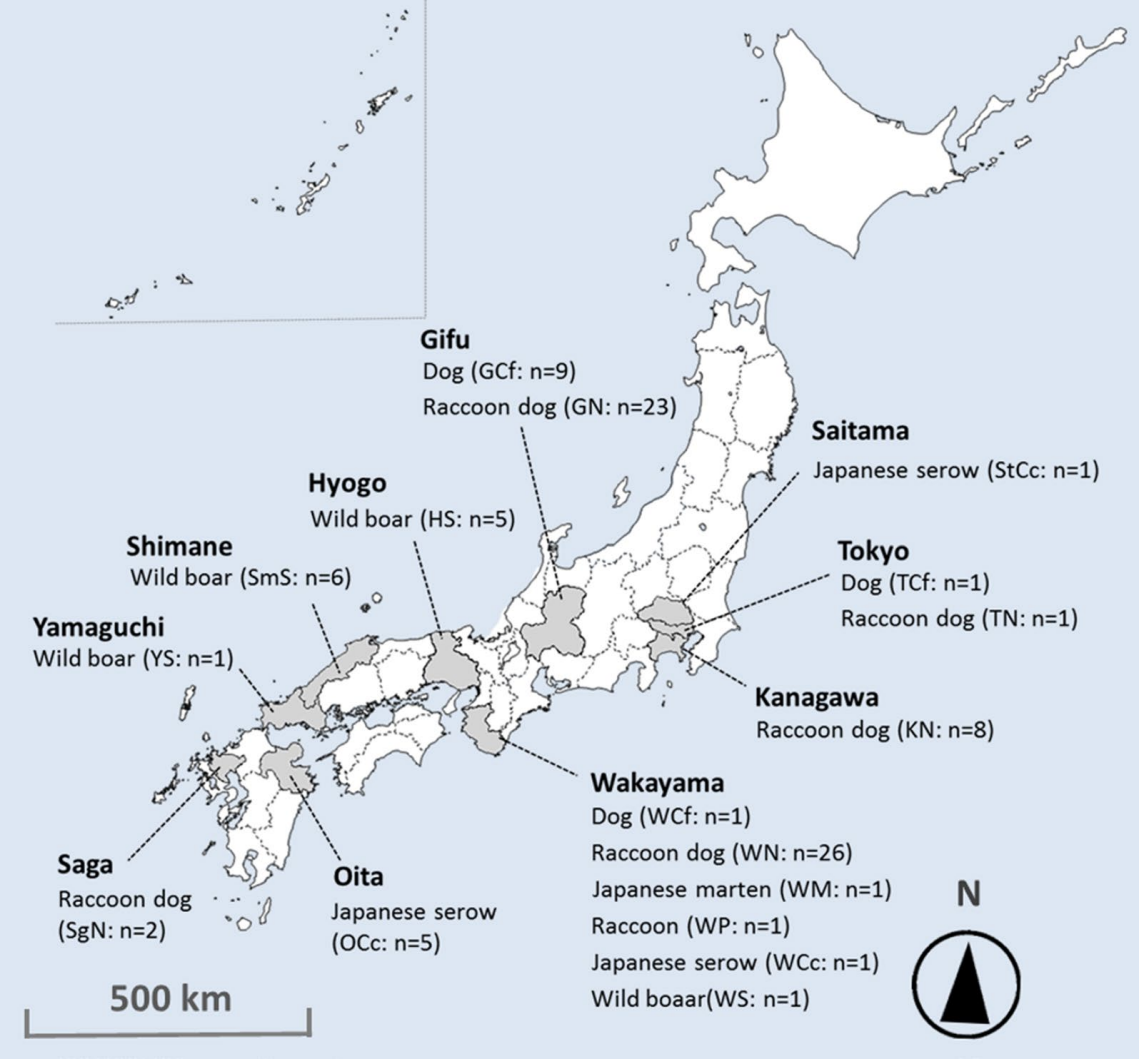
The location of the 10 sampling areas and 93 sampled animals in the present study

From 1996 to 2016, we collected skin crusts from 93 mangy animals from 10 different prefectures in Japan: Tokyo, Kanagawa, Saitama, Gifu, Wakayama, Hyogo, Shimane and Yamaguchi on Honshu Island, and Oita and Saga on Kyushu Island (Fig. 1).

The animals belonged to 17 populations from 6 species that consisted of raccoon dogs (*n* = 60), domestic/companion dogs (*n* = 11), raccoons (*n* = 1), Japanese martens (*n* = 1), Japanese serows (*n* = 7) and wild boars (*n* = 13) (Table 1). All skin crusts were stored at – 30 °C or in 70% ethanol until the mite collection. We collected one *Sarcoptes* mite per individual animal by the postponed (post-frozen) isolation method for frozen skins proposed by Alasaad et al. [32]. All mites were confirmed to be *S. scabiei* from the morphological criteria defined by Fain [10].

**Table 1:** Host species, higher-level taxon and the number of mite samples in each area

Prefecture	Host species	Host taxon	Sample size	Sampling year	Population name
Tokyo	Domestic dog	Carnivora (Caniformia)	1	2012	TCf
	Raccoon dog	Carnivora (Caniformia)	1	2012	TN
Kanagawa	Raccoon dog	Carnivora (Caniformia)	8	2012–2014	KN
Saitama	Japanese serow	Cetartiodactyla (Ruminantia)	1	1996	StCc
Gifu	Domestic dog	Carnivora (Caniformia)	9	2014–2015	GCf
	Raccoon dog	Carnivora (Caniformia)	23	2007–2015	GN
Wakayama	Domestic dog	Carnivora (Caniformia)	1	2010	WCf
	Japanese marten	Carnivora (Caniformia)	1	2010	WM
	Raccoon dog	Carnivora (Caniformia)	26	2009–2014	WN
	Raccoon	Carnivora (Caniformia)	1	2010	WP
	Japanese serow	Cetartiodactyla (Ruminantia)	1	2010	WCc
	Wild boar	Cetartiodactyla (Suina)	1	2011	WS
Hyogo	Wild boar	Cetartiodactyla (Suina)	5	2011	HS
Shimane	Wild boar	Cetartiodactyla (Suina)	6	2011	SmS
Yamaguchi	Wild boar	Cetartiodactyla (Suina)	1	2011	YS
Oita	Japanese serow	Cetartiodactyla (Ruminantia)	5	1996, 2016^a^	OCc
Saga	Raccoon dog	Carnivora (Caniformia)	2	2009	SgN

^a^Only the OCc 5 was collected in 2016

### DNA extraction and fluorescence-based PCR analysis

After the crushing individual mites, the DNA of each *Sarcoptes* mite was extracted using the DNeasy Blood & Tissue Kit (Qiagen, Tokyo, Japan). Molecular analyses were conducted following the method of Alasaad et al. [33] with slight modifications. The DNA from each mite was extracted and the 9 specific SSRs (Sarms 33, 34, 36–38, 40, 41, 44 and 45; Walton et al. [12]) from *Sarcoptes* mites were amplified by multiplex polymerase chain reaction (PCR). Each 5 µl PCR mixture contained 1 µl of the isolated mite DNA together with the PCR mixture containing all primer pairs (each primer ranged from 0.04 to 0.1 µM), 200 µM dNTPs, 0.5 µl of 10× PCR buffer (200 mM KCl, 100 mM Tris-HCl, pH 8.0), 0.5 mM MgCl_2_ and 0.05 µl (0.5 U/reaction) Ex Taq (TaKaRa Shuzo, Kyoto, Japan). Fluorescent PCR amplicons were analyzed in an ABI PRISM 3130 Genetic Analyzer using GeneScan 500 Liz® Size Standard (Applied Biosystems, Foster City, CA, USA). Allele calling was performed using the GeneMapper® Software v.4.1 (Applied Biosystems, Foster City, CA, USA).

### Molecular analysis

Observed (H_O_) and expected (H_E_) heterozygosity, linkage disequilibrium (LD) and Hardy–Weinberg equilibrium (HWE) tests were calculated using GENEPOP v.4.0. [34]. In this software, the exact *P*-values of LD tests were given by Chi-square tests and those of HWE were calculated by a Markov chain algorithm [34].

While some of the analyzed mite populations significantly deviated from HWE, LD was also detected between some SSR loci. Therefore, we used two methods that do not require assumptions, like HWE and linkage equilibrium (LE) of loci, to analyze the underlying population genetic model. First, to describe differentiation between mite populations, principal components analysis (PCA) was carried out using the package *adegenet* [35] of R v.3.2.2 [36]. Multivariate ordination in the PCA does not require data regarding the HWE and LD [37]. In this analysis, genetic relationships among mites associated with 17 host populations (Table 1) were analyzed. Secondly, to analyze the genetic relationships between mites, we calculated genetic distances (proportion of shared alleles; D_SA_ [38]) among mites using Populations v.1.2.32 [39]. These genetic distances were then visualized using the NeighborNet network provided by SplitsTree4 [40].

However, HWE in populations and LE between SSR loci are often violated in natural populations and are viewed as unrealistic [37]. In such circumstances, Bayesian clustering analysis by STRUCTURE software v.2.3.4 [41] has often also been employed for populations deviated by HWE and/or for loci in LD (e.g. Rasero et al. [15]). Thus, we applied the STRUCTURE analysis to elucidate the genetic structure of 93 *Sarcoptes* mites and to support the former 2 analytical methods. We performed 100,000 MCMC (Marcov Chain Monte Carlo) simulations followed by 50,000 steps of burn-in for each K value (K = 1–17), and these calculations were repeated independently 10 times. Subsequently, the most likely clustering number of K was determined by the peak of ΔK calculated by the method of Evanno et al. [42]. The website STRUCTURE HARVESTER (taylor0.biology.ucla.edu/structureHarvester) [43] was used for this calculation. The results of 10 replicate runs for each value of K were averaged using the Greedy algorithm of CLUMPP v.1.1.2 [44], and clustering results for each value of K were displayed graphically using distruct v.1.1 [45].

## Results

One hundred and eight alleles from the 9 SSR loci of all 93 mites were detected (Table 1); they contained 41 alleles from 11 domestic/companion dog-derived mites (TCf, GCf and WCf), 66 from 60 raccoon dog-derived mites (TN, KN, GN, WN and SgN), 10 from a marten-derived mite (WM), 12 from a raccoon-derived mite (WP), 25 from 6 serow-derived mites (StCc, WCc and OCc), and 61 from 13 boar-derived mites (WS, HS, SmS and YS). The number of alleles for each locus ranged from 7 (Sarms 36) to 18 (Sarms 33). H_O_ and H_E_ ranged from 0.152 (Sarms 44) to 0.337 (Sarms 37) and from 0.501 (Sarms 38) to 0.881 (Sarms 33), respectively.

A significant deviation from HWE was observed for some loci in *Sarcoptes* mite populations associated with KN, GN, GCf, WN, HS, OCc and SmS (*P* < 0.05, see Additional file 1: Table S1), and LD was confirmed between Sarms 33 and Sarms 34 (*χ*^2^ = 26.14, *df* = 12, *P* = 0.010), Sarms 34 and Sarms 40 (*χ*^2^ = 31.11, *df* = 8, *P* < 0.001), Sarms 34 and Sarms 45 (*χ*^2^ = 30.08, *df* = 12, *P* = 0.003), Sarms 40 and Sarms 41 (*χ*^2^ = 19.81, *df* = 8, *P* = 0.011) and Sarms 40 and Sarms 45 (*χ*^2^ = 15.61, *df* = 9, *P* = 0.048), when tested in the whole population.

**Fig. 2:**
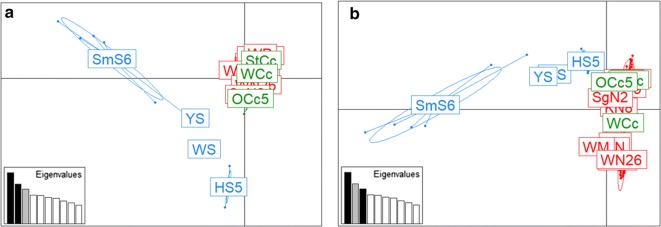
Results of the principle components analysis (PCA) of mite populations associated with 17 host populations. PCA showing the genetic structure of 93 mites from 17 host populations with component 1 (explaining 9.96% of the variance) *versus* component 2 (7.68%) (**a**) and component 3 (6.76%) (**b**). The eigenvalues of the two axes are displayed in each graph. Caniformia-, serow- and boar-derived mites are represented as red, green and blue, respectively. Each Host-associated mite population is indicated in the centre of component mites. Abbreviations for populations are provided in Table 1

The results of the PCA are shown in Fig. 2. The projected inertia of components 1, 2 and 3 were 9.96, 7.68 and 6.76%, respectively. In components 1 and 2 (cumulative projected inertia: 17.65%), Caniformia- (TCf, TN, KN, GCf, GN, WCf, WM, WN, WP and SgN) and serow-derived mites (StCc, WCc and OCc) formed a cluster and were separated from boar-derived mites (WS, HS, SmS and YS). In components 1 and 3 (cumulative projected inertia: 16.73%), Caniformia- and serow-derived mites were slightly more scattered than components 1 and 2, but formed a cluster, and were separated from boar-derived mites (Fig. 2).

**Fig. 3:**
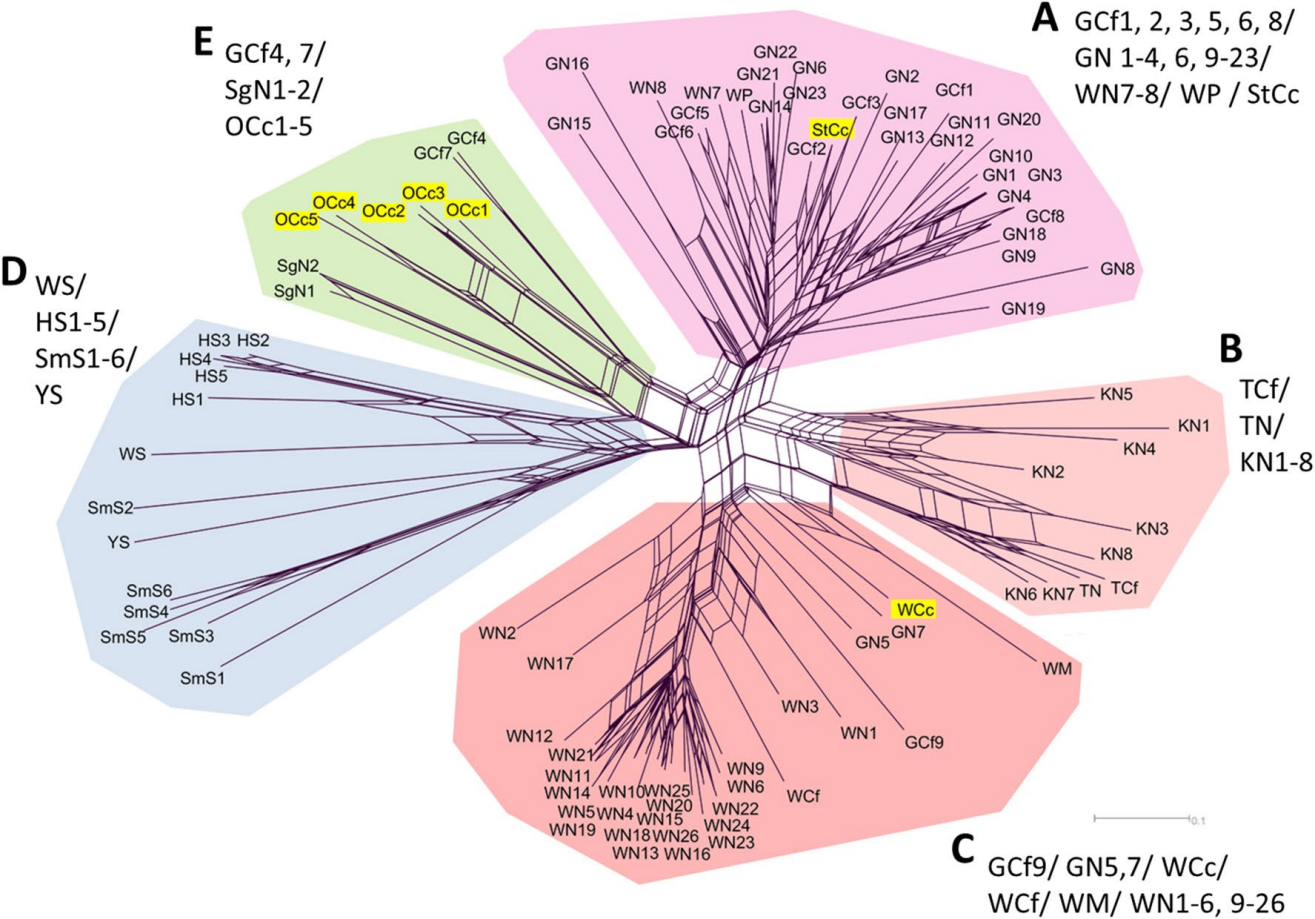
NeighborNet network constructed by D_SA_ between each pair of 93 mites. Main clusters (A, B, C, D and E) are separated by different colors. The genetic differentiation between clusters B and C is not clear, and WM, WCc, GN5, GN7 were experientially assorted into Cluster C. Japanese serow-derived mites (StCc, WCc, OCc1-5) are shown with the yellow background. Abbreviations for populations are provided in Table 1

The NeighborNet network composed by D_SA_ showed the 5 genetic clusters (Clusters A–E) in 93 mites (Fig. 3). Basically, mites from the same taxonomic or close host species within the same area were included in each cluster; Cluster A comprised Caniformia-derived mites from Gifu (GCf and GN), Cluster B comprised Caniformia-derived mites from Tokyo (TCf and TN) and Kanagawa (KN), Cluster C comprised Caniformia-derived mites from Wakayama (WCf and WN), Cluster D comprised boar-derived mites (WS, HS, SmS and YS) and Cluster E comprised serow-derived mites from Oita (OCc). However, some host species of other areas and/or different host taxa were also classified in each cluster. Raccoon- and raccoon dog-derived mites (WP1, WN7 and WN8) from Wakayama, and a Japanese serow-derived mite from Saitama (StCc) were grouped in Cluster A. A domestic/companion dog-derived mite from Gifu (GCf9) was grouped in Cluster C. Raccoon dog-derived mites from Saga (SgN) and domestic/companion dog-derived mites from Gifu (GCf4 and GCf7) were grouped in Cluster E. Raccoon dog-derived mites from Gifu (GN5, GN7), a Japanese serow-derived mite and a Japanese marten-derived mite from Wakayama (WCc, WM) showed just intermediate genotypes between Clusters B and C (we included them in Cluster C for the convenience in the discussion). All Japanese serow-derived mites were confirmed to be close to the Caniformia-derived mites.

The STRUCTURE analysis revealed the highest value of ΔK at K = 9 (Additional file 2: Figure S1). At K = 9, a similar result to that obtained using the NeighborNet network was seen, wherein the wild boar-derived mite population and Caniformia- with Japanese serow-derived mite population were assigned to different clusters. Subsequently, a second run of STRUCTURE analysis was performed for the 80 mites from the Caniformia- with Japanese serow-derived population, and the ΔK value showed the uppermost hierarchical cluster to be K = 4. The genetic structure in these 80 mites in K = 4 was also consistent with that observed using the NeighborNet network (Fig. 3 and Additional file 2: Figure S1).

## Discussion

As previously described, Rasero et al. [15] comprehensively presented the genetic differentiation of *Sarcoptes* mites in mammals in western Europe. Nonetheless, we have to re-attempt the interpretation of the results of their study here, before going into the profound discussion of our results. In our opinion, the results presented by Rasero et al. [15] lack accuracy because of the ambiguous definitions of terms related to “host taxon” (i.e. “carnivore”, “omnivore” and “herbivore”). Although Rasero et al. [15] did not define “host taxon,” the words “carnivore,” “omnivore,” and “herbivore” are usually used in the context of the foraging ecology of animals. In their study, the red fox, stone marten (*M. foina*) and pine marten (*M. martes*) were defined as “carnivores.” These animals are widely recognized as omnivores based on their food habits [46, 47]. Therefore, if these animals were classified by their food habits, their sorting would be incorrect. Thus, more accurately, the differentiation of *Sarcoptes* mite populations observed in their study did not conform to the foraging ecology of mammals, but to the taxonomy at the suborder level: (i) Caniformia in Carnivora as a substitute for “carnivore”; (ii) Suina in Cetartiodactyla as a substitute for “omnivore”; and (iii) Ruminantia in Cetartiodactyla as a substitute for “herbivore”.

As far as the present study is concerned, the taxonomy of our target species was as follows: domestic dog, raccoon dog, raccoon and Japanese marten belong to the suborder Caniformia in Carnivora; wild boar belongs to the suborder Suina in Cetartiodactyla; and Japanese serow belongs to the suborder Ruminantia in Cetartiodactyla. Deficiency of observed heterozygosity and deviation from HWE in some of the SSR loci in a few mite populations were also confirmed in the previous reports of other *Sarcoptes* mite populations [15, 16, 18, 20]. This phenomenon is due to a lack of random mating of mite populations between host animals, because *Sarcoptes* mites do not have free-living stages and individual hosts function as their ephemeral habitats in patchy environments [33]. Bearing in mind the presence of deviations in HWE, though a PCA method which will not be influenced by those genetic biases existed, we obtained the following consistent results in the multiple analyses: (i) the close genetic relationship between the mite populations derived from hosts in the same taxon, represented in omnivorous Caniformia and in wild boar populations; (ii) the separation of genetic clusters among mites derived from omnivorous Caniformia and from wild boar populations; and (iii) the close genetic relationship between mites derived from Caniformia species and from Japanese serow against the difference of host taxon. The results (i) and (ii) correspond to the previously reported genetic relationships of *Sarcoptes* mite populations in the European mammalian fauna and are consistent with the “Host–taxon phenomenon” formulated by Rasero et al. [15]. Meanwhile, (iii) shows the deviation from the Host–taxon regulation in the transmission web. Those serow-derived mites collected in the middle of 1990s (StCc1 in 1996 and OCc1-4 between 1996 and 1998) and in the 2010s (WCc1 in 2011 and Occ5 in 2016) from different locations highly suggested the long-time maintenance of gene flow between Caniformia- and serow-derived mite communities at several distant locations.

In the previous studies, the prey-predator pattern was described as “Curse of prey” [18] and tended to focus on the one-directional infections of *Sarcoptes* mites from the herbivorous preys to their carnivorous predators. However, this one-directional concept is not supported in case of “very weak” prey-predator relationships, the epidemiological history and the ecology of Japanese serow. Rather, infection from Caniformia mammals to serows is highly suspected. Upon reviewing the recent epidemiological history of sarcoptic mange in Japan, it was seen that its outbreaks in Japanese serows started roughly ten years later than the epidemic in raccoon dogs [21]. From a questionnaire survey for the epidemiology of sarcoptic mange in wild mammals, observations of mangy cases in hunting as well as in wildlife rescue practices began in the 1970s and 1980s in raccoon dogs and other Caniformia species [21, 22]. In the mid-1990s, recordings in hunting and rescue cases in raccoon dogs had drastically increased [21]. During this time, cases of sarcoptic mange in the serows increased [21, 25]. It might be possible that the epidemic in serows was associated with the increase of mangy cases in raccoon dogs (and possibly other Caniformia species).

From the view point of Japanese serow ecology, which is a territorial and monogamous species, usually living solitarily [48], the transmission of sarcoptic mange between serows has been considered to be less frequent than that in mammals which exhibit strong sociality (i.e. making herds or family groups) such as the raccoon dog, domestic dog and wild boar [2, 49]. However, multi-locational outbreaks in serows were almost simultaneously recognized since the 1990s in several geographically distinct populations on the Honshu and Kyushu Islands of Japan [21]. This concurrent emergence of mange among distinct serow populations was possibly due to transmission from other Caniformia harboring *S. scabiei*. Based on the data obtained from a questionnaire survey conducted by the Wildlife Management Office [21], most of the epidemic areas of mange infection in Japanese serows overlapped with those of mange infection in raccoon dogs.

Given that the inter-species transmission of *Sarcoptes* mites arises through both direct or indirect contact [8], there are two likely explanations for the transmission from Caniformia populations to the serow populations: (i) the direct transmission upon touching the carcasses of mangy animals; and (ii) indirect transmission through the sharing of habitat intensively used by both mammal populations. For the direct contact, the probable reason is the exploring or foraging behavior for dead animals sometimes observed in ruminant species such as cattle [50] and deer [51]. There is no observation, but likelihood that serows behave similar to dead or debilitated mangy Caniformia animals. For the indirect contact, the common use of the same location (or microhabitats) by these species has often been recorded by sensor cameras [52]. Although the survival of *Sarcoptes* mites off the host is only a few days in the natural environment [8], infection through contaminated environments (e.g. animal trails, vegetation, soil, etc.) is not impossible [8, 53]. Further study on the *Sarcoptes* mite’s ecology and contact between host species, besides the transmission experiments, may furnish a clear answer for the observed gene flow between herbivore ruminants and Caniformia species.

Lastly, our data reveal that some *Sarcoptes* mites from Caniformia species and Japanese serows do not belong to dominant genotypes in the Caniformia-serow derived communities in their locations (Fig. 3 and Additional file 2: Figure S1). If we assume that the results of the present study represent the gene pool of *Sarcoptes* mites in Caniformia mammals and Japanese serows in Japan, it may imply the migration of *Sarcoptes* mites, which is perhaps associated with (i) natural events of host migration and dispersion, or (ii) artificial translocation of *Sarcoptes* mites. Note that GCf4 and GCf7 from the domestic/companion dogs in Gifu belonged to the mite group from raccoon dogs and serows in Kyushu Island (OCc1-5 from Oita and SgN from Saga). Kyushu and Gifu are geographically distinct (> 550 km) and separated by sea. Thus, it is unlikely that this genetic relationship was formed by a natural event. Rather, this link could probably be owed to the artificial translocation of mangy animals, such as domestic/companion dogs. However, there are, of course, possible alternative hypotheses for the observed close genetic relationships between distant *Sarcoptes* mite populations, such as (i) an effect of a small sample size in mite populations between the two distant mite populations (i.e. limited observation and un-detection of similar genotypes), and (ii) a chance effect of mutation and convergence of genetic characteristics. Future research is needed for understanding of this type of genetic relationships and also the possible artificial impact for the expanding distribution of sarcoptic mange.

## Conclusions

The results of our study show that in Japan, *Sarcoptes* mite populations follow a “Host–taxon” regulation between wild boars and Caniformia with Japanese serow communities but deviate between the Caniformia and Japanese serow. To our knowledge, this result provides the first genetic evidence for a possible hidden transmission of sarcoptic mange between host species belonging to different taxa without unambiguous predator–prey systems. In the diverted transmission patterns of *S. scabiei* among multi-host systems worldwide, there may be cryptic relationships of *S. scabiei* populations in local mammalian communities. Although the effect of sarcoptic mange in wildlife species is still poorly understood [54], the understanding of the cryptic transmission of sarcoptic mange in local mammalian communities is necessary for the conservation of remnant and fragmented populations of wild mammals. In fact, sarcoptic mange is considered as one of the causes for the population decline in a remnant Japanese serow population on the Kyushu island of Japan [25]. For the preservation of vulnerable populations of Japanese serows and their related *Capricornis* species, sarcoptic mange infection from the Caniformia species, in addition to the possibility of artificial translocation of mangy animals (i.e. domestic/companion dogs), should be deliberated in wildlife management programs.

## Additional files

**Additional file 1: Table S1.** Results of Hardy–Weinberg equilibrium tests for each microsatellite locus for each Host-associated *Sarcoptes* mite population.

**Additional file 2: Figure S1.** Population structure as inferred by STRUCTURE analysis of *Sarcoptes* mites in the present study. **a** The graph for K = 9 with the 93 mites. **b** The graph for K = 4 with 80 mites (Caniformia- and Japanese serow-derived mites, excluding wild boar-derived mites from 93 mites). Each mite genotype is represented by a single vertical bar plot. Each color represents one cluster, and the proportion of each color in each bar plot shows the likelihood of assignment in the inferred clusters. Mite populations associated with each host animals are separated by thick black lines. 1, TCf; 2, TN; 3, KN1-8; 4, GCf1-9; 5, GN1-23; 6, WCf; 7, WM; 8, WN1-26; 9, WP; 10, SgN1-2; 11, StCc; 12, WCc; 13, OCc1-5; 14, WS; 15, HS1-5; 16, SmS1-6; 17, YS. For host population abbreviations see Table 1.

## Data Availability

Data supporting the conclusions of this article are included within the article and its additional files. Raw data are available from the corresponding author upon reasonable request.

## Abbreviations

HO: observed heterozygosity
HE: expected heterozygosity
HWE: Hardy–Weinberg equilibrium
LD: linkage disequilibrium
LE: linkage equilibrium
MCMC: Marcov Chain Monte Carlo
PCA: principal components analysis
PCR: polymerase chain reaction
SSR: simple sequence repeat
