# Supplementary material for: Possible transmission of Sarcoptes scabiei between herbivorous Japanese serows and omnivorous Caniformia in Japan: a cryptic transmission and persistence?
Source: Parasit Vectors. 2019 Aug 5;12:389. doi: 10.1186/s13071-019-3630-5 (PMC6683528; doi:10.1186/s13071-019-3630-5)
Supplement: Supplementary file 1 — Additional file 1: Table S1. Results of Hardy–Weinberg equilibrium tests for each microsatellite locus for each Host-associated Sarcoptes mite population. [file 13071_2019_3630_MOESM1_ESM.docx]

**Additional file 1: Table S1.** Results of Hardy-Weinberg equilibrium tests for each microsatellite locus for each host-associated *Sarcoptes* mite population.

| **Host population** | **Sarms 33** | **Sarms 34** | **Sarms 36** | **Sarms 37** | **Sarms 38** | **Sarms 40** | **Sarms 41** | **Sarms 44** | **Sarms 45** |
| --- | --- | --- | --- | --- | --- | --- | --- | --- | --- |
| TCf | Na | Na | Na | Na | Na | Na | Na | Na | Na |
| TN | Na | Na | Na | Na | Na | Na | Na | Na | Na |
| KN | 0.513 | 0.402 | 1.000 | 0.049* | 0.006** | Na | 0.007** | 0.008** | 0.024* |
| StCc | Na | Na | Na | Na | Na | Na | Na | Na | Na |
| GCf | <0.001** | 0.031* | 0.061 | 0.339 | 0.057 | <0.001** | <0.001** | 0.002** | 0.004** |
| GN | <0.001** | 0.005** | 0.075 | 0.004** | 0.009** | 0.662 | 0.533 | <0.001** | 0.019* |
| WCf | Na | Na | Na | Na | Na | Na | Na | Na | Na |
| WM | Na | Na | Na | Na | Na | Na | Na | Na | Na |
| WN | <0.001** | <0.001** | <0.001** | 0.001** | 0.044* | <0.001** | 0.081 | 0.020* | 0.003** |
| WP | Na | Na | Na | Na | Na | Na | Na | Na | Na |
| WCc | Na | Na | Na | Na | Na | Na | Na | Na | Na |
| WS | Na | Na | Na | Na | Na | Na | Na | Na | Na |
| HS | 0.015* | Na | Na | 1.000 | 0.032* | Na | Na | Na | Na |
| SmS | 0.030* | 0.031* | 0.094 | 0.167 | 0.091 | 0.005** | 0.010 | 0.138 | 0.315 |
| YS | Na | Na | Na | Na | Na | Na | Na | Na | Na |
| OCc | Na | 0.332 | 0.047* | 0.333 | Na | Na | Na | 0.333 | 0.110 |
| SgN | Na | Na | Na | 1.000 | Na | Na | Na | Na | Na |

Na: Not applicable, due to small sample size or lack of allele diversity, **P* < 0.05, ** *P* < 0.01
