# Supplementary figures and images for: Possible transmission of Sarcoptes scabiei between herbivorous Japanese serows and omnivorous Caniformia in Japan: a cryptic transmission and persistence?
Source: Parasit Vectors. 2019 Aug 5;12:389. doi: 10.1186/s13071-019-3630-5 (PMC6683528; doi:10.1186/s13071-019-3630-5)

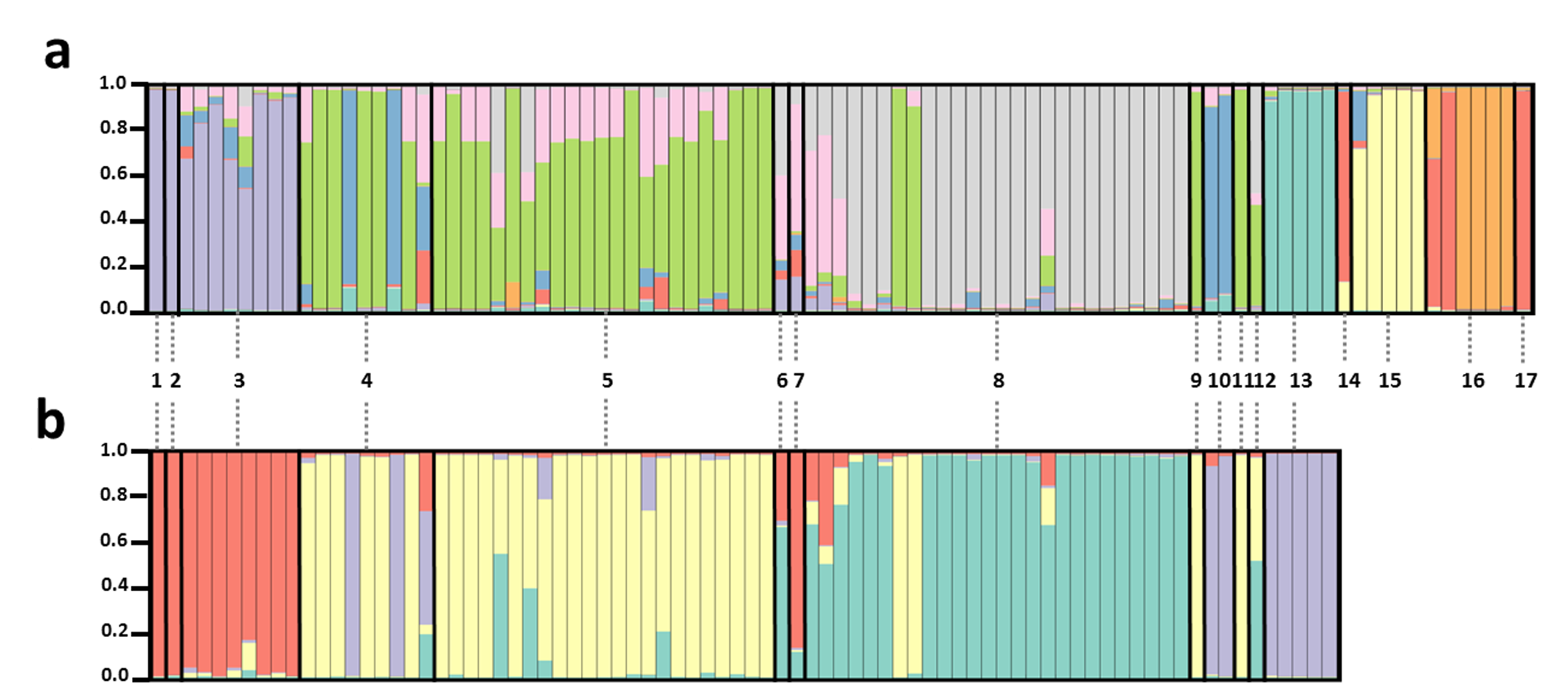

Supplement: Supplementary file 2 — Additional file 2: Figure S1. Population structure as inferred by STRUCTURE analysis of Sarcoptes mites in the present study. a The graph for K = 9 with the 93 mites. b The graph for K = 4 with 80 mites (Caniformia- and Japanese serow-derived mites, excluding wild boar-derived mites from 93 mites). Each mite genotype is represented by a single vertical bar plot. Each color represents one cluster, and the proportion of each color in each bar plot shows the likelihood of assignment in the inferred clusters. Mite populations associated with each host animals are separated by thick black lines. 1, TCf; 2, TN; 3, KN1-8; 4, GCf1-9; 5, GN1-23; 6, WCf; 7, WM; 8, WN1-26; 9, WP; 10, SgN1-2; 11, StCc; 12, WCc; 13, OCc1-5; 14, WS; 15, HS1-5; 16, SmS1-6; 17, YS. For host population abbreviations see Table 1. [file 13071_2019_3630_MOESM2_ESM.tiff]
